# Coparenting in English-Speaking and Chinese Families: A Cross-Cultural Comparison Using the Survey Tool CoPAFS

**DOI:** 10.3390/children10121884

**Published:** 2023-12-01

**Authors:** Tianmei Zhu, Marsha Kline Pruett, Jonathan Alschech

**Affiliations:** 1 Department of Human Development and Family Studies, University of Connecticut, Storrs, CT 06269, USA; tianmei.zhu@uconn.edu; 2 School for Social Work, Smith College, Northampton, MA 01060, USA; jalschech@smith.edu

**Keywords:** coparenting, cross-cultural studies, Chinese, factor analysis, couple communication

## Abstract

While coparenting-related conceptual frameworks and empirical studies have received considerable attention in Western countries, there is far less attention on this topic in other regions. This study seeks to fill this gap by comparing coparenting dynamics between English-speaking and Chinese parents. This study begins by reviewing coparenting relationships in both Western and Chinese contexts. Study participants comprised 399 English-speaking parents living in the US and Canada and 534 Chinese parents living in Mainland China. There were several waves of participant recruitment by sending out the flyers online or utilizing the professional networks to invite eligible parents. The measurement tool CoPAFS (Coparenting across Family Structures), which has been validated in English-speaking culture, was used to compare the differences in coparenting constructs in two cultures. First, the model fit of CoPAFS within Chinese culture was examined with Cronbach Alpha values and relevant model fit indices such as Comparative Fit Index and Root Mean square Residual. As most of the statistics fell below the expected level of excellence, there is a need to locally adjust the entire model in order to better interpret Chinese parenting. The intensity of connection between each factor included in the model and the coparenting relationship as a whole was then investigated. Although most factors were endorsed similarly by Chinese and English-speaking parents, there were notable differences in their opinions regarding communication and trust. While English-speaking parents highly valued these two elements within the coparenting process, Chinese parents showed almost no attentiveness to them. In order to understand factors that may contribute to such a sharp contrast, two main variables, culture and gender, were tested. Through a series of multigroup invariance analyses assessing equivalence across groups, it was discovered that culture emerged as the more dominant determinant among the groups of participants. The implications of cross-cultural use of the CoPAFS tool and future research directions are discussed.

## 1. Introduction

### 1.1. Definition and History of Coparenting Research

Over the past few decades, the coparenting relationship has become a prominent area of study, with scholars and practitioners alike debating the determinants of relationship quality as well as how individuals can play a crucial role in building and maintaining strong coparenting partnerships. As family systems theory posits, the whole family system encompasses interrelated subsystems in which family members interact in ways that cannot be fully understood by looking at dyadic relationships alone (e.g., mother–child or mother–father relationships) [1]. Acting as two managers with full agency responsible for parenting activities, parents need to acknowledge and respect the other’s practice during contingencies and crises of parenthood accompanied by likely tensions and frustrations [2,3]. One definition of coparenting acknowledges coparents as at least two individuals who share childrearing responsibilities upon mutual agreement [2]. The practice involves parents’ joint cooperation that divides childrearing duties, including instrumental support (e.g., financial) and emotional support after the birth of a child.

Some of the first empirical studies focus on triadic family relations, which examined parental interaction in analyses of the frequency and intensity of parents’ supportive or undermining behaviors of each other, as well as how parents negotiate and strategize coparenting [4,5]. Built upon these observations, consequent studies identified three major conceptual domains of coparenting: (1) the amount of conflict between parents regarding child-rearing issues and overall household rules, (2) the extent to which parents support, value, and respect each other, and (3) triangulation, which involves the formation of coalitions between one parent and the child to undermine the involvement of the other parent [6]. This model was then expanded, specifying joint family management, support/undermining, childrearing agreement, and division of labor as its four main components [2]. Two years later, in response to this model, scholars broadened the traditional definition of parenting beyond heterosexual family units and claimed that coparenting encompasses diverse coparent identities and family structures regardless of individuals’ sexual orientation or relationship status [3]. They also argued that flexibility is essential when defining equality in parenting and determining appropriate forms of it, as coparents’ values and beliefs, which are often influenced by cultural and environmental factors, greatly shape what constitutes coparenting to them. A coparent may be absent for a time but still has representational meanings for the other coparent and the child [3]. The family system is dynamic, with interparental interaction, including coparenting support and conflict, fluctuating throughout children’s early adolescence [7].

Compared to Western countries, research on coparenting in China and neighboring areas began relatively late. The first article mentioning the term ‘coparenting’ was published in 2004 in Taiwan, focusing on a sample of 309 couples raising preschool children [8]. Additional studies contributed to an initial wave of coparenting research in Mainland China. In addition to embracing the shared definition of coparenting that has been derived from mostly Western research, Chinese scholars incorporated Chinese-specific elements for the localization of the concept and its research application. Deepening the research in reference to models suggested by Western professionals such as the ecological model of coparenting and supportive and undermining coparenting behaviors within the multi-layered family system, Chinese scholars discussed some phenomena observed in Chinese cultures and explained them with cultural factors [5,9,10]. For example, different from the typically defined collaborative or hostile behaviors in English culture, “red face and white face” is a term used to describe a more blurred coparenting pattern in Chinese families. In this pattern, one coparent adopts a strict and firm parenting style, while the other coparent takes on a more nurturing and caring role, with the aim of better disciplining and educating their children [11]. “Strict father, kind mother” is a specific manifestation of this coparenting strategy that is influenced by traditional gender roles [12].

### 1.2. Assessing Coparenting

As psychologists have endeavored to expand the boundaries of knowledge in this field, the need for standardized scientific measures of coparenting relationships have become substantial. Several widely used psychometric measurement scales were devised to define the potential components of assessing and evaluating the interparental relationship. McHale’s 16-item Coparenting Scale covers four factors within family relationships: family integrity, disparagement communication, conflict in the child’s presence, and coparental disciplinary activities. Out of the four factors, family integrity and disciplinary activities are the positive coparenting behaviors, and disparagement communication and conflict are the negative ones [13]. Eleven items involve “overt and family-level behaviors”, while the other five items focus on “covert one-to-one activities” [14]. By capturing expanded coparenting concepts (e.g., the words of affirmation between parents in the presence of the child), the scale presented a diverse range of family intercourses beyond direct parenting behaviors for the child, emphasizing the affection between parents [14] (p. 4). In his later research, McHale added coparenting conflict and cohesion as two additional observable variables. Trained coders observed and coded the videotaped records of assessments of parents and interpreted the data to assess these two variables [15].

Feinberg and his colleagues also developed scales for coparenting. Based on a series of exploratory factor analyses and reliability analyses for Feinberg’s Family Foundations program, the researchers transformed their theoretical conceptualization to three subscales that assess Coparental Support, Parenting-Based Closeness, and Coparental Undermining. Later, as subdomains of support and undermining emerged, the researchers created scales to measure Coparental Agreement, Exposure of the Child to Conflict, Endorsement of Partner’s Parenting, and Division of Labor as four other domains [16,17,18]. The Coparenting Relationships Scale (CRS) offers a total score of seven subscales for the overall quality of the coparenting relationship in addition to subscale scores. All the domains and the total score exhibit strong internal consistency across parental gender and time points, ranging from 0.61 to 0.94 [18]. Sometimes certain subscales were extracted and regrouped to measure positive and negative coparenting as well [19]. The scale was later translated and revised in other cultures such as Italian- and Portuguese-speaking cultures. In China, the original seven subscales were shortened to six and the items were also revised [20].

The Coparenting Inventory for Parents and Adolescents (CI-PA) relied on self-reports of parents and children [21]. The inventory consists of cooperation, conflict, and triangulation as three dimensions of coparenting. However, instead of considering younger children like infants or toddlers, adolescents and their parents were the subjects being studied. The researchers examined the reliability and validity of the scale. The convergence between parents was satisfactory while convergence between parents and their children was weaker yet still statistically significant. The analysis supported the salience and necessity of all three dimensions in regard to coparenting.

Some typical types of coparenting patterns assessed with the use of these scales include cooperative families and conflictual families. While the former represents connectedness and higher functioning, the latter represents more distress and disagreements [22,23]. Some other coparenting patterns, though less commonly mentioned in the literature, indicated high levels of cooperation and conflicts, or disconnected families with low levels of the same [22,23].

However, even though coparenting scales have been developing for many years with various measures created based on different criteria, none of these scales are applicable across all family structures and cultures. For this study, we employed a coparenting scale that we had previously developed, which was designed to be more adaptable and flexible. This scale was developed by examining relevant literature and combining and condensing items from previous instruments to capture the most important factors outlined in prior scales. Through the refinement of the emergent themes, some common factors were identified across prior research. The items included in these factors were factor analyzed in exploratory analysis, testing the internal validity of factor structure assembled in different ways until five distinct factors were identified in the coparenting scale used for the current study: trust, communication, animosity, valuing the other parent, and respect.

### 1.3. Five Factors

#### 1.3.1. Trust

As most definitions agree, trust consists of people’s expectation that another’s actions will be beneficial, favorable, or at least not detrimental to them [24,25,26]. The way in which people trust others is largely dependent and shaped by their existing experience of being attached to others, especially early caregivers. Secure individuals feel trust due to previous positive trust-related memories and the ability to set proper relationship goals and strategies in close relationships [27,28,29,30]. They also tend to be more willing to commit and build interdependence [31,32]. A higher level of trust plays the vital role in romantic relationships of reducing negative couple interpersonal outcomes and facilitating positive ones. In particular, trust serves as a mediator between attachment orientations and relationship outcomes [33]. It was found to mediate the relation between female attachment anxiety/male attachment avoidance and relationship satisfaction [34]. The trust a mother has for her partner mediated her attachment avoidance and coparenting relationship quality [33]. A causal relationship was also discovered between destiny beliefs and forgiveness, where individuals experiencing the state of attachment anxiety with stronger destiny beliefs are slower to show forgiveness towards their partners [35]. With partner-specific trust mediating the causal relationship, the higher level of trust leads to a greater likelihood of forgiveness. The research indicated that trust was a significant mediator in explaining an additional 34% of the variability in co-parenting conflicts and 35% of the variability in parenting alliances.

#### 1.3.2. Valuing the Other Parent

The degree to which coparents are committed to coparenting is influenced by their mutual feelings towards each other [36]. In the process of making plans and taking care of the children, coparents may encounter differences in their perspectives and preferences. As two coparents witness their child’s behaviors, they may pursue different approaches to guide the child’s development by either actively intervening or going with the flow. However, the interparental dynamics can be supportive as long as both parents recognize, acknowledge, and value the potentially distinct qualities inherent in the other parent’s character or discipline beliefs. Conversely, if one coparent assumes more responsibility for child-rearing and possesses more information and authority about the children, they may engage in restrictive gatekeeping, which refers to the difficulty of relinquishing part of the coparenting responsibilities to the other coparent [36].

Proper endorsement of another parent’s parenting involves people’s positive attitudes and behaviors towards their partner regarding childrearing issues [18]. This affirmation can be physical or verbal, in the presence of the child or not, as reflected in everyday life [14]. When both coparents value each other’s role in raising their children, it fosters an atmosphere of collaboration and helps to minimize conflict. The positive effect of parents working together is particularly evident in stressful situations, where reciprocal interparental support and agreement leads parents to work together to solve problems [37,38]. The active involvement of multiple caregivers helps each caregiver effectively manage daily challenges in childrearing. In the context of everyday life, maintaining a positive coparenting relationship can benefit the entire family, resulting in fewer behavioral problems in children and serving as an indicator of both parents actively contributing to parenting as a unified team [39].

#### 1.3.3. Respect

In order to establish and maintain a successful co-parenting relationship, it is crucial for partners to collaborate closely and form a strong parenting alliance [2,40]. The idea of the parenting alliance highlights the importance of partners’ perceptions and feelings about the quality of their co-parenting relationship, rather than just their interactions. Even if partners communicate well and make joint decisions, their perceptions and attitudes toward each other can still have a significant impact on the success of their co-parenting relationship. Respect is an important but often overlooked aspect of this alliance, as it goes beyond just being polite and courteous to the other person and requires actively valuing and being open to the thoughts and behaviors of the other person [41]. In romantic relationships where power balance is sought, respect is commonly associated with traits such as trustworthiness, love, care, and acceptance, and is generally associated with emotional warmth and reciprocity [42]. Respect is a core value in marital relationships along with commitment, intimacy, and forgiveness and typically accompanies feelings of admiration and fondness for a partner [43,44]. Although it is not defined similarly across writings and studies, empirical studies have demonstrated that respect is closely related to the level of commitment partners have to their relationship, their ability to cope with relational trauma, their overall satisfaction with the relationship, and their willingness to engage in prosocial behaviors [45,46]. Insufficient respect received can be devastating to the maintenance of the coparenting relationship. It turned out fathers’ perception of bad coparenting can lead to their withdrawal from bearing parenting responsibilities. And the effect was observed to be less obvious in mothers [47].

#### 1.3.4. Communication

Coparents utilize both instrumental and affective communication to exchange information. Instrumental communication involves discussing basic life issues that require agreement, while affective communication involves sharing emotions and feelings. By employing these two types of communication consistently, parents support each other in response to both negative and positive events that arise in their daily lives. To manage challenging situations together, partners engage in dyadic coping, which entails both partners engaging in a dyadic appraisal of the circumstance and communicating about it with each other [24,48]. Coparents also capitalize on positive events to share happiness and enhance the benefits they experience [49]. Achieving such synchronization of coparents’ mental states, however, requires clear and direct communication. Clarity ensures the message conveyed is not vague or camouflaged; directness requires the message to be directly brought to the other coparent without any intermediaries [50]. Similar communicative styles also apply to effective parent–child conversations. The increased level of conversation orientation (more freedom to air disagreements and different ideas) is beneficial for children to align the expectations and experiences of parent–child relationships [51].

The benefit of effective communication is obvious. When one coparent communicates an event, it allows the other to respond, which can strengthen their bonds and attachments [24]. This aligns with the intimacy model proposed in 1988, in which self-disclosure and response is the foundation to satisfy both parties’ needs in the interpersonal exchange process, since both help to build intimacy in romantic relationships [29]. In fact, this set of exchanges was named “matching support” as “a request for advice followed by informational support or the disclosure of emotions followed by emotional support” [52] (p. 755). Providing matching support to the partner who seeks an active response was predictive of higher perceived partner sensitivity and higher marital satisfaction [52]. By learning about each other and reducing the gap between perceptions, coparents can deepen their understanding and connection, which in turn strengthens their relationship.

On the other side, negative communicative patterns within married couples can be devastating to the relationship. Four types of specific negative behaviors that increase marital conflict and may lead to divorce were listed in the relevant research piece— criticism, defensiveness, contempt, and stonewalling—labeled as The Four Horsemen of the Apocalypse [53]. Criticism involves complaining and attacking the person being criticized. Defensiveness entails an ineffective self-protective response in a conversation that can be a counterattack. Contempt causes psychological harm to the partner through the expression of disgust and disrespect. Stonewalling occurs when one withdraws from conversation and distances themselves from the partner.

#### 1.3.5. Animosity

Animosity is a key factor that induces conflicts between parents and dysfunctional interpersonal dynamics. It is characterized as a negative global assessment of another partner “who is judged to be deserving of no respect as a parent” [54] (p. 404). Various forms of animosity such as global criticism, corrosive contempt, shielded defensiveness, and passive stonewalling all interfere with normal parenting negotiations and can lead to accumulated stress, frustration, and emotional distress for both parents in the long term [50,54]. This massive spiral eventually builds up, escalating negative reciprocity within the relationship. As the partner’s guardedness becomes more severe, it can result in discouragement towards the other party or even deprivation of opportunities for the other person to spend time with the children. This impact is particularly pronounced in men, as their reported depression is largely influenced by coparenting experiences shaped by their female partner [55].

Animosity can have a spillover effect on the parent–child relationship, as the presence of marital animosity experienced by either parent can negatively impact their partner’s relationship with the child [56,57]. This can create a conflict-ridden parenting environment, with parents deflecting and redirecting their anger toward their children, resulting in less interaction, responsiveness, and withdrawal from children, and an increase in the use of stricter control or punishments [4,58,59]. The toxic high-conflict interparental dynamics can have a detrimental impact on the functioning of all the other relationships within the family system.

#### 1.3.6. Studying Coparenting beyond the Western Scope

While coparenting research has made significant progress in developed Western countries, there is a significant lack of knowledge about what coparenting and family relationships mean to the other parts of the world, particularly China. A large number of Chinese scholars have focused on grandparent–grandchild intergenerational coparenting instead of parental coparenting in which parents directly bear most of the coparenting responsibility. An integrative intergenerational coparenting framework was invented, encompassing dimensions beyond the traditional aspects of parenting measurement, for example, power and authority [60].

This trend took place because of the unique structural challenges Chinese families have faced over time. In rural areas of China, many parents opt to work in cities for higher salaries to support their family, leaving their children to be cared for by grandparents who reside in their hometowns. In cities, since the price of housing continues to climb, parents usually have to live with grandparents because they themselves cannot afford an apartment [61,62]. These culture-specific features create a three-generation family structure in most parts of China, which impedes Chinese scholars from directly applying Western theories about nuclear families to researching Chinese families. A 2017 national survey found that over 80% of grandparents directly participate in childcare activities for their grandchildren, often assuming parental responsibilities [63]. Due to the prevalence of the phenomenon of raising children across multiple generations, most scholars focus on intergenerational parenting. In contrast, coparenting between parents seems less common as a form of childrearing in China and has not gained sufficient attention from researchers. The lack of research on Chinese nuclear families has negative implications that extend to the field of social work. Limited data and results make it challenging for scholars to advocate for policy changes that directly target these families and promote family welfare [64].

Even as some scholars bring attention to parenting in nuclear families, the meaning and implications of parenting styles may differ in China because of culturally specific beliefs derived from Taoism and Confucianism [65,66,67]. Chinese parents often base their parenting behaviors on the traditional concept of training children to temper their willpower and cultivate their perseverance, which typically involves a higher degree of devotion and strictness [66]. Parental strictness can be viewed as a display of both concern and caring, depending on which of the two forms it manifests: parental control (negative) or parental organization (positive) [65,68]. As long as the parent’s parenting behavior fits into the universal framework of the authoritative parenting style (high levels of warmth, acceptance, and responsiveness to children’s demands), it is believed to have a positive impact on the overall development of Chinese children, resulting in fewer internalizing [69,70,71,72].

To understand each culture’s coparenting philosophy at a deeper level, sociocultural lenses are necessary to provide insight into the environment which shapes patterns of thinking and behaving. Rather than simply emphasizing research on parent–child interactions or couple relationships, which overlook parenting context, the inclusion of coparenting helps target interparental relationships and produces meaningful results to inform potential future directions in cross-cultural coparenting research. Until now, Chinese and English literature have explored coparenting separately, with minimal intersection and cross-cultural comparison, despite the fact that scholars have accumulated rich knowledge about coparenting within their respective countries or areas. This study aims to bridge the gap between these two cultures and stimulate conversation on coparenting ideologies that are shared or divergent among parents from the other side of the world. Through preliminary exploration, this study aims to establish a significant foundation for guiding future cross-cultural research efforts in this area. This research will not only inform the analytical strategies needed for comparing Chinese and English cultures but also for any other two cultures.

#### 1.3.7. The Present Study and the Research Questions

In the present study, we examine the validity of the coparenting measurement tool CoPAFS (Coparenting Across Family Structures), which contains five factors, to test the feasibility of applying it in a Chinese context. Furthermore, we utilize this tool to compare differences in coparenting ideologies across English and Chinese cultures with a specific focus on married couples. The data analysis quantitatively accounts for differences in the strength of associations between English-speaking and Chinese parents, and furthermore explores the key determinants causing such differences.

## 2. Materials and Methods

This study consists of four questions:

1. Does the measurement model underlying the CoPAFS scale exhibit a good fit with the data gathered from Chinese parents?
2. What are the differences in the relative importance of each factor, in terms of each of the factor loadings on coparenting as the latent construct, compared between the English-speaking parents and the Chinese parents?
3. Does gender significantly account for the variation on coparenting as well each of the five factors measured by the CoPAFS scale?
4. Does culture significantly account for the variation on coparenting as well each of the five factors measured by the CoPAFS scale?

In respect to each of these research questions, we offer the following hypotheses.

**H1:**  *The CoPAFS measurement model exhibits a good model fit on the data collected from married Chinese parents.*

**H2:**  *The relative importance of the factors, in terms of factor loadings on coparenting as the latent construct, varies significantly between the English-speaking parents and the Chinese parents.*

**H3:**  *Gender significantly accounts for the variation on the CoPAFS scale as a whole and on each of the five factors, both for English-speaking parents and for Chinese parents.*

**H4:**  *Culture significantly accounts for the variation on the CoPAFS scale as a whole and on each of the five factors, both for English-speaking parents and for Chinese parents.*

### 2.1. Participants

To be eligible for participation in this study, the participants needed to be (1) a parent with a child under the age of 18 at the time of completing the survey; and (2) a parent who was practicing shared parenting with at least one other adult. Since the focus of this study is on married couples, only Chinese and English-speaking parents who identified as married at the time of answering the survey were included in the final dataset; separated or unmarried parents were excluded from this study. As there were several iterations of CoPAFS surveys collected in North America, married English-speaking parents who answered the survey in any of three iterations were included in the final dataset for analysis. Thus, while data on Chinese-speaking parents were collected in the context of a single survey, which opened in 2022 February and closed in October 2022 (8 months in total), data on English-speaking parents were collected in three separate iterations, of which only the last version exactly parallels the Chinese one. The only difference between the iterations was in how demographic questions were phrased, while the phrasing of the CoPAFS scale items remained identical.

The sample eventually comprised 348 English-speaking mothers, 51 English-speaking fathers, 437 Chinese mothers, and 97 Chinese fathers. The first English-speaking sample consisted of 153 parents, including 132 married mothers and 21 married fathers. The second sample consisted of 139 married mothers in total. The third sample consisted of 77 married mothers and 30 married fathers. The age range most commonly reported by the Chinese parents was between 30 and 39 years old, with the overall sample ranging between 20 years or younger to 50 years or older. The age range of English-speaking parents was from 20 to 50 or older and they were more likely to fall into either younger or older age groups. Specifically, the percentage of English-speaking parents aged between 20–29 and 50 or older in each of the three English-speaking groups was higher than the percentage of Chinese parents in those same age groups. Compared to Chinese parents, two out of the three English-speaking groups consisted of significantly more individuals over the age of 50; the other one English-speaking group had a significantly higher proportion of participants aged between 30–39.

Most Chinese parents reported earning a bachelor’s degree. Similarly, most English-speaking participants reported having a bachelor’s degree. One out of the three English-speaking groups was more specialist-level-educated than Chinese parents and another English-speaking group had significantly more individuals with university-level education.

Most of Chinese parents also self-identified as working class, with upper middle class comprising the second largest group. The economic status of English-speaking participants was distributed more evenly across the different socioeconomic groups. Two out of the three English-speaking groups have significantly more upper-class individuals than Chinese parents, and one English-speaking group has significantly more lower middle-class individuals. Demographic tables are provided below. The total number of responses to the demographic questions did not add up to the total number of participants because a few people left a few items blank, but these omissions were negligible. Table 1, Table 2 and Table 3 show the detailed characteristics of the participants.




**English-Speaking Parents**

**(1)**

**Economic Status**

**Frequency**

**Percent (%)**
<USD 20,00032.0USD 20,000–39,00021.3USD 40,000–59,00063.9USD 60,000$−79,000 159.8USD 80,000 or more12078.4Missing74.6Total153100Note: Because of slight inconsistency of income standards between the first wave of data and the subsequent two waves, the first sample is listed separately here.


### 2.2. Instruments

**Figure 1:**
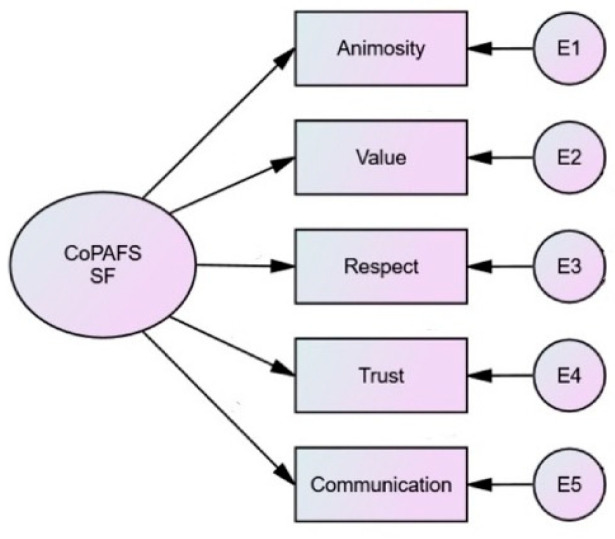
Measurement model for CoPAFS.

Coparenting was measured using the short form 27-item version of Coparenting Across Family Structures Scale (CoPAFS-27) which was developed by Michael Saini and Marsha Kline Pruitt and validated in their follow-up study published in 2019 [73]. Figure 1 shows the structure of the model.

The scale comprises 5 subscales: Animosity, Value (Valuing the other parent), Trust, Respect, and Communication, and is intended to measure coparenting dynamics across all family structures. The 27 items are each categorized into one of the five subscales and were measured by a 5-point Likert scale ranging from 1 = Strongly Disagree to 5 = Strongly Agree. All items were originally developed in English. Then, they were translated by one Mandarin native speaker and were reviewed and fine-tuned by a Mandarin-speaking research assistant and a sociology professor at a leading Chinese university with expertise in the field of coparenting. The triple check process ensured the accuracy of the statements and their cultural adaptations due to linguistic subtleties. When arranging all the items on the survey, the order was randomly shuffled so items would not cluster together around each factor.

In the original survey, some questions are positively phrased while some are negatively phrased. A strong agreement with a high score on some questions may refer to a low level of the attribute being measured, while other questions are measured in the reverse way. To have all the directions of the questions consistent for later addition of the total scores, the scores on questions 3, 5, 7, 8, 10, 11, 13, 15, 17, 18, 20, 22, 24, 25, and 27 were reversed (5 became 1, 4 became 2, 3 stayed 3, 2 became 4, and 1 became 5). Table 4 shows the full list of questions below.

### 2.3. Data Collection Procedure

The survey was circulated among parents in China and in North America. English-speaking parents were recruited through multiple online platforms such as Facebook, Instagram, parenting organizations, and some personal networks of our colleagues. In the case of Chinese parents, in addition to these listed resources, we also sent out flyers to WeChat groups and WeChat public official accounts. Our Chinese colleague also referred us to some local non-profit organizations she collaborated with as well. An online version of the survey was offered to the participants, which took them around 30 min to complete once they gave the informed consent at the beginning of the survey. The participants were informed about their rights to inquire about the research or express any concerns, as well as the potential risks and how to contact the researchers and/or Smith College IRB. The whole process protected participants’ anonymity and their data’s confidentiality. Personal information was neither requested nor collected, and responses were not shared with any external sources in full observance of the protocol approved by the Smith College IRB.

### 2.4. Data Analysis Strategies

We utilized 11 V29 and its extension SPSS AMOS V28 to complete all the necessary statistical analysis. To test the stability of the short-form CoPAFS factor structure, the Chinese sample was divided into 2 sub-samples: (1) Chinese mothers; (2) Chinese fathers. For each group, the psychometric properties of the short-form scale and the 5 subscales around which the 27 items were hypothesized to cluster were calculated to test the validity of the short-form 27-item CoPAFS in its Mandarin translation, as it was found to perform well in English-speaking samples in the previous study. The internal consistency of each of the 5 subscales and the short-form CoPAFS scale as a whole was expressed in terms of the Cronbach’s Alpha coefficients.

Confirmatory factor analyses (CFA) were conducted with a maximum likelihood estimation method for each of the 5 subscales on both Chinese mothers and fathers, which assessed how well the measurement model captured the covariance between the items composing the 5 factors. In terms of the whole scale, the analyses showed how well the measurement model captured the covariance between the 5 subscales. Multiple model fit indices were calculated and reported. First, a chi-squared test indicating the difference between observed and expected covariance metrics was calculated. However, as the value is strongly influenced by the sample size, this could be misleading in either small samples (leading to acceptance of an inappropriate model) or large samples (leading to rejection of appropriate models). A sample size above 200 cases may result in non-significance even when the model is appropriate [74]. Second, the Root Mean square Residual (RMR) showing the square root of the discrepancy between the sample covariance matrix and the model covariance matrix was calculated. Third, the Comparative Fit Index (CFI) representing the discrepancy between the data and the hypothesized model was calculated, while adjusting for issues of sample size inherent in the chi-squared test of model fit. Fourth, the Root Mean Square Error of Approximation (RMSEA) measuring the discrepancy between the hypothesized model with optimally chosen parameter estimates and the variance matrix was calculated and reported.

The analysis also estimated the regression coefficients for each factor and the proportion of the variation explained by the factors relative to the whole model, providing information about the stability of the construct across groups—the extent to which the subscales and the whole scale measure what they were intended to measure. Thus, it helped determine the comparability and accuracy of the model when applied to different groups. Last, a set of multigroup invariance analyses were conducted to determine whether gender or culture—or both—were significantly related to variation on the coparenting scale as a whole and on each of the five factors. With the data collected from each group, invariance analysis helped test the stability of the factor structure of the CoPAFS scale and the individual parameters for equivalence across groups with conditions being gradually constrained. Configural, metric, and scalar invariance were tested. Configural invariance revealed if the same general specification of the model holds across the groups of participants and exhibits a good fit across groups while allowing the parameters of the unconstrained model within each group to be freely estimated; metric invariance added further equality constraints to structural covariances; scalar invariance constrains the measurement residuals in addition to the previous conditions.

## 3. Results

### 3.1. Model Fit Examination of CoPAFS Scale Using the Data Collected from Chinese Parents

#### 3.1.1. Cronbach Alpha Values and the Correlation Matrices for Five Factors

Given that the internal validity of the CoPAFS model for English-speaking families was already examined and demonstrated in a previous study, the first part of the results will solely report the Cronbach Alpha coefficients and model fit indices for Chinese families [73]. The Cronbach Alpha coefficient, which estimates the extent to which the items in the scale are measuring the same underlying concept, yielded a value of 0.925 for the entire CoPAFS scale in married Chinese families, on a scale from 0 to 1. Such a high value indicates an excellent level of internal consistency of this scale to measure the coparenting relationship of Chinese families as a closely related set of items. However, the Cronbach Alpha coefficients for each factor vary considerably, ranging from quite satisfactory high values to low ones, indicating poor internal consistency as indicated in Table 5 below. Trust, animosity, and communication exhibited high Cronbach Alpha coefficient values, implying the participants’ response values across the subsets of questions were very consistent. However, valuing the other parent and respect showed low values, implying loose inter-relations across items within these two subscales.

Beyond the investigation of internal consistency of the single subscales, correlations between the factors were also calculated to explore overlaps between the different subscales. Table 6 indicates moderate to high positive correlations between most factors, suggesting that the subscales may measure similar concepts. It is important to note, however, that these findings may also be attributed to the closely clustered nature of family-related concepts in people’s minds, which might make it difficult for individuals to clearly differentiate between these five dimensions.

#### 3.1.2. Model Fit Indices

For Chinese mothers, the chi-squared value of the whole model was 352.981 (df = 5, *p*-value < 0.01), which is high for the differences between observed and expected covariance metrics. However, the value of $x_{m}^{2}$ is overly sensitive to the sample size when testing the effectiveness of a model across different populations [75,76]. Therefore, there is no specific range of chi-squared values that can be considered universally good or bad. The Root Mean square Residual (RMR) was 0.887, the square root of the difference between sample and model covariance matrix. A value less than 0.08 is generally acknowledged as indicative of a good model fit. After adjusting for the sample size representing the chi-squared value, the Comparative Fit Index (CFI) was calculated to be 0.762, which is lower than the common acceptable cutoff value of 0.9. This value demonstrates the degree of discrepancy between the data and the model that was hypothesized. The Root Mean Square Error of Approximation (RMSEA) value was 0.399, which manifests the difference between the hypothesized model using the best parameter estimates and the variance matrix. However, this value is greater than 0.08 as the acceptable cutoff value. For Chinese fathers, the value of the chi-squared test was 101.288 (df = 5, *p* < 0.01); the RMR value was 1.04; the CFI value was 0.718; the RMSEA value was 0.448.

These model indices served as continuous indicators of the model–data correspondence and could be interpreted as “goodness or badness of fit” of the model. Though some indices are quite sensitive to sample size, others are not. After combining the indices obtained from both Chinese mothers and fathers and evaluating them, we found that both patterns were similarly suboptimal. The CoPAFS model as a whole did not appear to be a particularly strong fit for the data, indicating that it may require further refinement in future studies in order to improve its ability to accurately replicate the English data for Chinese married families.

Generally, the statistics refute the first hypothesis. This information is valuable for future research as efforts are needed to improve and refine the model until adequate model fit can be demonstrated. Yet, additional information gleaned from the analyses suggests a more complicated picture than a simple rejection.

### 3.2. The Relative Importance of Each Factor as Endorsed by English-Speaking Parents and Chinese Parents

Factor Loading Analyses

We next explored the strength of the relationships between each factor and the latent variable, the coparenting relationship, across all sample groups. A higher value indicates a stronger association of the factor in accounting for the proportion of variance on the latent construct. The maximum value for both the regression coefficient and variance of the coparenting relationship itself was set to 1 (Table 7).

The regression coefficients of each subscale for Chinese mothers ranged from 0.98 to 0.00, with animosity having the highest coefficient of 0.98, trust as 0.86, valuing the other parent as 0.75, respect as 0.57, and communication with 0.00 as the lowest. All the results are statistically significant (*p* < 0.001) except for communication (*p* = 0.36). The results showed that Chinese mothers are most attentive to animosity and least attentive to communication while evaluating the coparenting relationship. The proportions of the variation of each subscale accounted for on the underlying construct were calculated along with the regression coefficients. Animosity explained 96.7% of the variance; trust explained 74.4%; valuing the other parent explained 57.2%; respect explained 32.7%; and communication explained 0.0%.

The pattern for Chinese fathers was nearly identical to that of mothers. The highest regression coefficient was 0.97 for animosity, then 0.90 for trust, 0.72 for valuing the other parent, 0.52 for respect, and −0.03 for communication. Animosity explained 94.5% of the variance; trust explained 82.1%; valuing the other parent explained 53.1%; respect explained 27.1%; and communication explained 0.0%. Again, all the results were statistically significant (*p* < 0.001) except for communication (*p* = 0.27). According to these regression coefficients, the communication factor did not explain any of the variation in the coparenting relationship quality for both Chinese mothers and Chinese fathers.

Among Chinese parents, the understanding of a good coparenting relationship specified by the five subscales was quite different than in Western culture. Trust and respect had the same highest correlation coefficients of 0.89 among English-speaking mothers. Then, the correlation was 0.87 for animosity, 0.86 for communication, and 0.71 for valuing the other parent. Trust and respect both explained 80.3% of the variance; animosity explained 77.2%; communication explained 74.7%; and valuing the other parent explained 50.6%. All the results are statistically significant (*p* < 0.001).

A similar hierarchy of the importance of the subscales was found in English-speaking fathers. Trust has the highest coefficient of 0.97; respect is 0.87; communication is 0.83; animosity is 0.82; and valuing the other parent is 0.81. All the results are statistically significant (*p* < 0.001). Trust explained 87.0% of the variance; respect explained 76.9%; animosity explained 76.2%; communication explained 70.4%; and valuing the other parent explained 66.5%. All five subscales were important to English-speaking parents commenting on their coparenting relationships.

The major contrast between English-speaking parents and Chinese parents’ attitudes was the importance ascribed to respect and communication. Chinese parents tended to place less emphasis on respect as a factor contributing to a healthy coparenting relationship, while communication was even deemed to be almost irrelevant in Chinese culture. In contrast, both respect and communication were significantly more influential in the opinions of English-speaking parents and received the same credit as the three other factors. Given all of the statistics above, the second hypothesis was accepted because there were structural differences in the strength of correlations between English-speaking and Chinese couples.

### 3.3. Testing the Significance of Gender and Culture as Two Predictors That Account for the Variation in Coparenting and the Five Factors

Multigroup Invariance Analysis

As slight differences in gender and the disparate gap in regressions between respect and communication and coparenting relationship were observed between the two cultures in the second research question, the importance of gender and culture as factors that explain noteworthy differences was investigated. Controlling for either gender or culture, four multigroup invariance tests of the model were run, each testing for configural, metric, and scalar invariances. In the general measurement model, three covariances between value and communication, respect and communication, and value and respect were present and acknowledged for more accurate outcomes. The invariance was tested between Chinese mothers and fathers, English-speaking mothers and fathers, Chinese mothers and English-speaking mothers, and Chinese fathers and English-speaking fathers. Results are summarized below in Table 8, Table 9, Table 10 and Table 11.

Table 8 compared Chinese mothers and fathers to see if gender would be a predictor of significant differences in Chinese culture. At the configural level, CFI is 0.99 and RMSEA is 0.05, which showed the great fit of the model in terms of configural arrangement across Chinese mothers and fathers. At the metric level which constrained structural covariance, the chi-squared value is not significant (*p* = 0.66) and the null hypothesis, that there was equivalence in factor variances and covariances between the two groups, was accepted. Moving to the most restrictive scalar level, the chi-squared value was still non-significant (*p* = 0.22). The fit of the model was ensured with the invariance of measurement residuals. At all three levels of invariance analysis, there was no significant variance between Chinese mothers and fathers. Thus, it was concluded that gender was not a significant predictor of the variation on the coparenting scale as a whole nor on each of the five subscales for Chinese parents.

Table 9 compared English-speaking mothers and fathers. At the configural level, CFI is 0.93, which is good, while RMSEA is 0.14, which is weak. The statistics showed the model is acceptable in terms of configural arrangement across English-speaking mothers and fathers. At the metric level, the chi-squared value is not significant (*p* = 0.38), and the null hypothesis was accepted, indicating that there was equivalence in factor variances and covariances between the two groups. Moving to the most restrictive scalar level, the chi-squared value was still non-significant (*p* = 0.02). The fit of the model was ensured with the invariance of measurement residuals. At all three levels, we found only significant invariance between English-speaking mothers and fathers. Thus, similarly to Chinese parents, it was concluded that gender was not a significant predictor of the variation on the coparenting scale as a whole, nor on each of the five subscales in English-speaking parents.

Overall, neither Chinese nor English-speaking parents illustrated distinct differences explained by their gender. In response to the third research question, gender was not found to be a significant predictor of the variation on coparenting. The hypothesis to the third research question was hence rejected.

Next, two multigroup invariance tests were conducted to assess culture as a potential predictor while holding gender constant. For both tests, the CFI and RMSEA values were not ideal. CFI did not reach the benchmark of excellence while RMSEA was higher than the normally accepted value, suggesting the model fit was inadequate. Consequently, null hypotheses at both metric and scalar level were rejected, claiming that the structure of the model significantly differed between Chinese and English-speaking mothers. It became apparent that culture was a significant predictor of variation in the coparenting scale and its five subscales, for mothers across both cultures. In the process of accumulating profiles for the coparenting relationship, culture carried more weight than gender.

Comparing Table 10 and Table 11, the results of the comparison of the two father groups differed in comparison to the mother groups: the metric invariance was accepted but not the scalar invariance. Taking measurement weights and structural covariance into account supported that there is no significant variance of the model between Chinese and English-speaking fathers. However, once restraining the measurement residuals, significant differences appeared, which is the same as mothers’ scalar-level invariance comparison. Strictly speaking, the result still showed a significant cultural effect on fathers’ values towards the five factors.

Using the strictest level of multigroup invariance analysis, results indicated that culture was a statistically significant factor associated with differences in coparenting across groups (Chinese mothers vs. English-speaking mothers and Chinese fathers vs. English-speaking fathers). The findings suggest that the weight of each factor that influences coparenting relationships may differ significantly depending on the cultural context in which one responds to the items. Thus, the fourth hypothesis that highlights the importance of culture was accepted.

## 4. Discussion

### 4.1. Research Overview

Built upon the review of the theoretical framework and measures constructed by other scholars in the coparenting field, the CoPAFS scale was developed to expand research on families in different contexts such as diverse family structures or cultures [14,16,18]. In the past few years, English-speaking parents with different marital statuses were used to validate the CoPAFS scale in North America, but the cross-cultural feature of the scale as part of the original goal has not been explored and examined until now [73]. In this study, the overarching objective is to identify similarities or differences between English-speaking and Chinese married parents in recognition of the factors constituting coparenting relationship wellbeing via this innovative measurement tool. We first estimated the fit of the CoPAFS model in the Chinese context. Then, we measured the strength of different factors in both cultures. With noticeable significant differences across groups, we further investigated whether gender or culture contributed to such variations.

### 4.2. Model Fit of CoPAFS under the Chinese Context

Although the CoPAFS model was proven to be a valid measurement for English-speaking participants, the application of the model did not demonstrate effectiveness in this pilot study in a Chinese context [73]. We first investigated how well the items are closely clustered within the whole scale and each subscale. The Cronbach Alpha value for the entire scale is 0.925, which represents quite a high internal consistency of the scale. However, values of certain factors, specifically valuing the other parents and respect, are low compared to information obtained from English-speaking parents, showing that items within these two factors are more loosely interrelated. As we further looked into various model fit indices generated from Chinese participants, the general conclusion we reached is that the overall model was a sub-optimal fit for the Chinese parents. The unsatisfactory results we obtained suggest that the current CoPAFS model is not yet an adequate representative measurement tool for the data from the recruited Chinese married parents and should not be immediately applied for probing Chinese parenting in real life.

The differences do not stem only from language differences, but from cultural differences as the Chinese sample emanated from China; they were not Chinese-speaking Americans. The model requires further refinement and localization before the researchers can use this tool to interpret the responses from Chinese parents accurately. To achieve this goal, a more theoretical understanding of the Chinese coparenting relationship is required for improving the scale. A larger sample, already under way, may better account for cultural nuances in coparenting among the Chinese parents.

### 4.3. Different Views of Chinese and English-Speaking Parents on Elements of Parenting

Moving from model examination to additional results, we used confirmatory factor analyses to test relationship strengths between each of the factors and the holistic coparenting relationship. The results illustrated statistically significant differences between Chinese and English-speaking parents in the weight they gave to communication and respect when evaluating the quality of their coparenting relationship. With the regression coefficients being 0.00 and −0.03% for Chinese mothers and fathers, Chinese parents showed a striking indifference towards the level of communication in coparenting. Much higher weight was given by English-speaking parents, with both regression coefficients hovering at approximately 85%. The limited appreciation for communication in Chinese coparenting seems contradictory to most Western literature that emphasizes the significance of communication in facilitating positive coparental conversation and coparents’ individual well-being [24,52]. A potential explanation pertains to gender-typed caregiving patterns that exist more or less in Chinese families [77]. There has traditionally been a greater emphasis on the parental role compared to the spousal role [78]. Other research supports that women tend to compartmentalize the distinct roles of being a parent versus being a spouse [79]. In most areas or countries, fathers traditionally are involved less in childcare than mothers [80]. While the proportion of fathers actively participating in childcare activities has increased in Western industrialized countries, Chinese fathers in general maintain their secondary provider role, which offers more financial than parenting support to meet the rigid social expectation of gender roles [81,82]. In contrast, Chinese mothers spend more time with children as emotional supporters and provide more practical care [82]. Some Chinese mothers express satisfaction with their role as caregiver and do not actively seek practical assistance from the child’s father in infant care [83].

This division of parenting responsibility forms an interesting phenomenon summarized by an old Chinese saying, “men work outside and women take care of the home”, which has gained renewed attention in China recently [84,85]. The tacit assumption of gender roles in Chinese culture may be a contributing factor to the lower perceived need for communication among Chinese parents. Due to the implicit division of parenting responsibilities based on gender, there may be less overlap in childrearing tasks for Chinese parents to discuss and reconcile. As a result, Chinese parents are more likely to rely on their own perceptions of responsibilities and complementary roles of the other parent when participating in the childrearing process [82].

In addition to communication, respect was also found to be of relatively less importance in the coparenting environment of Chinese parents. While the regression coefficients of respect are 0.57 and 0.52 for Chinese mothers and fathers, the same coefficients are 0.89 and 0.87 for English-speaking mothers and fathers. In comparison, Chinese parents valued respect nearly 30% less than English-speaking parents. The obvious difference in the respect domain between Chinese and English-speaking parents might be ascribed to the shared sociocultural environment that most Chinese participants experienced. The majority of them included in this study were urban residents born during the nationwide implementation of China’s One-Child Policy. The policy restricted the number of children married urban couples could have to one in order to control China’s population growth. There is a dramatic shift in personalities in the younger generation from their parents [86]. As the only child in the family, they were invested with more resources and tended to exhibit stronger individualistic characteristics and more open-mindedness towards entering marriage or not. Even if they are married, they are less tolerant of their partner’s different opinions [87]. These rigid views of parenting roles may lead to less effort or adherence to parental collaboration, regarding decisions and practices of the other parent, which in turn may contribute to their lower recognition of the importance of respect.

### 4.4. Culture as the More Dominant Predictor than Gender

Multigroup invariance analyses were used to explore whether gender or culture accounted for differences in weights of coparenting factors across four groups (Chinese fathers, Chinese mothers, English-speaking fathers, and English-speaking mothers). It was discovered that rather than gender, culture was related to more significant variation in the CoPAFS subscales. At the most restrictive scalar level of the analyses, significant variances were found between Chinese mothers and English-speaking mothers, as well as Chinese fathers and English-speaking fathers. Culture is a social construct that is fulfilled with sets of distinct beliefs and behaviors that are shared by people living in the same culture to regulate their behaviors [88]. Coparenting ideologies are learned behaviors that differ according to what is deemed normative in a particular culture. Even though the common goal shared by all parents across the world is to nurture and encourage their children to be competent in their society, the ways to achieve that goal vary greatly across cultural groups [89,90]. For example, there are significant variations in maternal behaviors across modern industrialized countries such as Argentina, Belgium, Israel, Italy, and the U.S. [88]. Additionally, levels of progressive parenting attitudes and modernity of childrearing attitudes vary considerably across countries as diverse as China, Colombia, Italy, Jordan, Kenya, the Philippines, Sweden, Thailand, and the U.S. [88]. Previous research has also compared attachment and self-regulation patterns of U.S. and Japanese mothers [88,91]. However, current literature on Chinese parenting mostly focuses on Chinese immigrant families in the U.S. rather than Chinese residents born in China. Thus, there is a lack of direct comparison between English-speaking and Chinese local cultures. This study provides a crucial piece of the puzzle for integrating cross-cultural parenting comparisons with empirical evidence that highlights the differences between these two cultures. Furthermore, this study serves as a resource for parenting scholars researching cross-cultural parenting styles, both conceptually and methodologically.

## 5. Limitations and Future Research

As mentioned earlier, it is crucial to ensure the CoPAFS scale’s model fits the Chinese culture to interpret the data accurately. To achieve cross-cultural validity of instruments, additional efforts are necessary to establish adapted equivalence [92]. Qualitative studies may be required to understand cultural and interpersonal dynamics among Chinese parents to enhance the instrument’s accuracy. It is also essential to confirm the conceptualization of profiles grouped under different factors to ensure that the measurement subscales have the same meaning across different cultural groups. Or, it may be fruitful to encourage analysis of different meanings ascribed across cultures to the concept of coparenting.

Regarding the methodology of this study, a set of multigroup invariance tests were used to calculate the ***p***-values, determining whether there were statistically significant differences by gender or culture. Given the sample size of nearly 1000 participants, statistically significant results could also be due to the large sample size [60].

Though the CoPAFS model shows some large differences in communication and respect between two cultures’ coparenting, the survey was completed by only 534 Chinese parents as the first wave of participants outside an English-speaking culture. There are likely geographical sampling limitations to this first study report. Moreover, the composition of participants recruited in this study are mostly middle-class and well-educated people, with a relatively low father participation rate compared to mothers. As a result, research findings may have low generalizability to populations characterized by different demographics.

For future research, there are a few directions that can be extended in further investigation. First, although this study showed a poor model fit of the CoPAFS scale for married Chinese parents, the model fit indices should be re-examined in other Chinese family structures, including those with mother–grandparent coparenting pairs. Second, this study did not account for the variance within English datasets, such as the racial and ethnic distribution of participants, as it focused on a broader culture-level comparison. However, future CoPAFS studies that focus on English-speaking populations might investigate racial diversity as an essential theme within and across English-speaking participants from differing cultural backgrounds.

## 6. Conclusions

This study tested the validity of the CoPAFS model and used it as a tool to reveal cross-cultural differences in coparenting constructs between Chinese and English-speaking married families. A series of model fit calculations revealed the currently limited applicability of the CoPAFS model in explaining Chinese parents’ responses. This finding challenged our initial hypothesis and highlights the need for future work to revise and enhance the cross-cultural adaptability of the scale. In light of this realization, this study ventured to compare parents from two distinct cultures, revealing a sharp contrast between the two in terms of the endorsement of respect and communication. The result substantiated our second hypothesis, which posits that the relative importance of the factors varies significantly between parents from two cultures. In order to examine the potential contributions of gender and culture, multigroup invariance analyses were conducted to test our third and fourth hypotheses. These analyses yielded novel evidence indicating that culture has a significant influence on the coparenting constructs endorsed by both Chinese and English-speaking parents, while the influence of gender was found to be less pronounced. As a result, the gender hypothesis was rejected, while the culture hypothesis was accepted. Overall, the findings of this study underscore the critical role of cultural context in the study of coparenting relationships and identifies promising new research directions for future studies employing the CoPAFS model.

There are a few practical as well as empirical implications in relation to this study. Although we have raised a popular gender-related childrearing pattern in China as one possible reason why communication receives almost no attention from Chinese parents, there is currently little Chinese literature directly addressing communication within the couple’s relationship. Using this gender-determined parenting theory to explain this cultural difference would be challenging without first confirming the connection between presumed work division and the low endorsement of communication. Therefore, further investigation is recommended in this area to delve deeper into this connection.

The same mechanism applies to the concept of respect. The One-Child Policy should only be considered to be just one of many social factors that contribute to the diminished significance of respect between parents. It may not be the most influential factor or, there could potentially be a mediating variable that directly impacts parents’ values. Therefore, conducting further research is necessary before a definitive and confident conclusion can be reached regarding the factors contributing to the downplaying of respect by Chinese parents.

Finally, the high endorsement of hostility among the participants raises interesting questions about the nature of marriages in China, and the role of conflict or anger in downplaying communication and respect. Perhaps the lack of communication helps keep the lid on covert hostilities that are intrapersonal and perhaps structurally created by conditions in Chinese society. Such questions await further research, with important implications for the well-being of China’s children and families.

## Figures and Tables

**Table 1:** Age distribution of the participants.

	Chinese Parents		English-Speaking Parents (1)			English-Speaking Parents (2)			English-Speaking Parents (3)		
Age	Frequency	Percent (%)	Frequency	Percent (%)	Significant (Y/N)	Frequency	Percent (%)	Significant (Y/N)	Frequency	Percent (%)	Significant (Y/N)
Under 20	1	0.2	0	0	N *p* = 0.84	0	0	N *p* = 0.84	0	0	N *p* = 0.84
20–29	34	6.4	13	8.5	N *p* = 0.19	12	9.5	N *p* = 0.19	9	8.4	N *p* = 0.24
30–39	238	44.6	64	41.8	N *p* = 0.73	75	59.5	**Y** ***p* = 0.03**	48	44.9	N *p* = 0.48
40–49	240	44.9	54	35.3	N *p* = 0.98	27	21.4	N *p* = 1.00	43	40.2	N *p* = 0.82
50 and older	20	3.7	20	13.1	**Y** ***p* < 0.01**	12	9.5	**Y** ***p* = 0.02**	7	6.5	N *p* = 0.13
Missing	1	.2	2	1.3		13	9.4		0	0	
Total	533	100.0	153	100		139	100.0		107	100.0	

**Table 2:** Education level distribution of the participants.

	**Chinese Parents**		**English-Speaking Parents** **(1)**			**English-Speaking Parents** **(2)**			**English-Speaking Parents ** **(3)**		
**Education Level**	**Frequency**	**Percent (%)**	**Frequency**	**Percent (%)**	**Significant (Y/N)**	**Frequency**	**Percent (%)**	**Significant (Y/N)**	**Frequency**	**Percent (%)**	**Significant (Y/N)**
Lower than high school	7	1.3	3	2.0	N *p* = 0.29	3	2.2	N *p* = 0.26	N/A	N/A	
High school/Post-Secondary	38	7.1	8	5.2	N *p* = 0.81	9	6.5	N *p* = 0.60	N/A	N/A	
Specialist	49	9.2	30	19.6	**Y** ***p* < 0.01**	9	6.5	N *p* = 0.86	N/A	N/A	
University	324	60.7	103	67.3	N *p* = 0.17	99	71.2	**Y** ***p* < 0.01**	N/A	N/A	
Others	113	21.2	0	0	N *p* = 1.00	0	0	N *p* = 1.00	N/A	N/A	
Missing	3	0.6	9	5.9		19	13.7		N/A	N/A	
Total	534	100.0	153	100		139	100		N/A	N/A	

**Table 3:** Economic status of the participants.

	Chinese Parents		English-Speaking Parents (2)			English-Speaking Parents (3)		
Economic Status	Frequency	Percent (%)	Frequency	Percent (%)	Significant (Y/N)	Frequency	Percent (%)	Significant (Y/N)
Poor	19	21.2	9	6.5	N *p* = 0.09	6	5.6	N *p* = 0.09
Working class	272	1.3	15	10.8	N *p* = 1.00	22	20.6	N *p* = 1.00
Lower middle class	86	7.1	12	8.6	N *p* = 0.99	26	24.3	**Y** ***p* = 0.03**
Upper middle class	149	60.7	30	21.6	N *p* = 0.94	22	20.6	N *p* = 0.95
Upper class	5	9.2	20	14.4	**Y** ***p* < 0.01**	31	29.0	**Y** ***p* < 0.01**
Missing	3	0.6	53	38.1		0	0	
Total	534	100.0	139	100		107	100	

Note: The categories of economic status have been adjusted according to different countries.

**Table 4:** Twenty-seven CoPAFS questions.

It is important that my child loves both parents	V
2. I value the other parent’s parenting skills	R
3. I feel awkward when I am with the other parent	A *
4. I work well with the other parent when decisions need to be made about our child	C
5. I am hostile or biting in my conversations with the other parent	A *
6. I can talk easily with the other parent about activities I would like to do with our child	C
7. I disagree with the choices that the other parent makes about our child	A *
8. I don’t think it is helpful to talk with the other parent about decisions that need to be made about our child	V *
9. I feel comfortable in sharing my thoughts about parenting with the other parent	C
10. I feel out of control when speaking with the other parent	A *
11. I find it difficult to support the other parent’s relationship with our child	T *
12. The other parent asks my opinion on parenting issues	C
13. My child would be better off seeing less of the other parent	V *
14. Although we don’t always agree, we respect each other’s differences as parents	R
15. I get little support from the other parent to help out with the work of parenting	T *
16. We parent better when we make decisions together	V
17. I have trouble controlling my anger when around the other parent	A *
18. I need to ‘go behind’ the other parent to fix the mess left behind	T *
19. When we meet face to face, the other parent and I are friendly or polite to each other	C
20. I pretend to support the other parent’s decisions but in the end, I do what I think is best for our child	T *
21. I trust the other parent with our child	T
22. I try to be more involved, but the other parent won’t let me have an opinion	R *
23. The other parent respects what I bring to parenting our child	R
24. I worry about my child while in the other parent’s care	T *
25. It is better to be away from, or uninvolved with, the other parent to make sure we don’t argue	A *
26. It’s important that the other parent is involved in our child’s life	V
27. The other parent tries to be a good parent but does not know enough about parenting to be the kind of parent our child needs	T *

In the right column, each letter corresponds to a factor name: T = Trust, V = Valuing the other parent, R = respect, C = communication, A = Animosity. * The asterisk sign represents the items being reversed.

**Table 5:** Cronbach Alpha for five factors.

	Cronbach Alpha	Number of Items per Factor
Value	0.50	5
Trust	0.86	7
Respect	0.30	4
Animosity	0.88	6
Communication	0.84	5

**Table 6:** Correlation matrices for the factors.

	Value	Trust	Respect	Animosity	Communication
Value	1				
Trust	0.704 **	1			
Respect	0.743 **	0.522 **	1		
Animosity	0.748 **	0.866 **	0.553 **	1	
Communication	0.354 **	0.081	0.513 **	−0.013	1

** *p* < 0.01.

**Table 7:** Proportion of variation accounted by each of the factors in four samples (regression coefficients).

	Animosity	Value	Respect	Trust	Communication
Chinese Married Mothers	0.98 *** (96.7%)	0.75 *** (57.2%)	0.57 *** (32.7%)	0.86 *** (74.4%)	0.00 (0.0%)
Chinese Married Fathers	0.97 *** (94.5%)	0.72 *** (53.1%)	0.52 *** (27.1%)	0.90 *** (82.1%)	−0.03 (0.0%)
English-speaking Married Mothers	0.87 *** (77.2%)	0.71 *** (50.6%)	0.89 *** (80.3%)	0.89 *** (80.3%)	0.86 *** (74.7%)
English-speaking Married Fathers	0.82 *** (76.2%)	0.81 *** (66.5%)	0.87 *** (76.9%)	0.97 *** (87.0%)	0.83 *** (70.4%)

*** *p* < 0.001.

**Table 8:** Multigroup invariance comparison: Chinese mothers vs. Chinese fathers.

Model	Chi-Square (*p* Value)	DF	CFI	RMSEA	ΔChi-Square	Δdf	ΔCFI	ΔRMSEA	Decision
M1-configural	19.86	4	0.99	0.05	N/P	N/P	N/P	N/P	N/P
M2-metric	20.27	9	0.99	0.04	0.41	5	0	0.01	Accept (p = 0.66)
M3-scalar	30.86	17	0.99	0.03	10.59	8	0	0.01	Accept (*p* = 0.22)

**Table 9:** Multigroup invariance comparison: English-speaking mothers vs. English-speaking fathers.

Model	Chi-Square (*p* Value)	DF	CFI	RMSEA	ΔChi-Square	Δdf	ΔCFI	ΔRMSEA	Decision
M1-configural	132.81	14	0.93	0.14	N/P	N/P	N/P	N/P	N/P
M2-metric	132.82	15	0.93	0.14	0.01	1	0	0	Accept (*p* = 0.38)
M3-scalar	146.17	20	0.92	0.12	13.35	6	−0.01	−0.02	Accept (*p* = 0.02)

**Table 10:** Multigroup invariance comparison: Chinese mothers vs. English-speaking mothers.

Model	Chi-Square (*p* VALUE)	DF	CFI	RMSEA	ΔChi-Square	Δdf	ΔCFI	ΔRMSEA	Decision
M1-configural	43.86	4	0.84	0.27	N/P	N/P	N/P	N/P	N/P
M2-metric	479.95	9	0.84	0.25	436.09	5	0	0.02	Reject (*p* < 0.01)
M3-scalar	735.28	17	0.76	0.23	255.33	8	0.08	0.02	Reject (*p* < 0.01)

**Table 11:** Multigroup invariance comparison: Chinese fathers vs. English-speaking fathers.

Model	Chi-Square (*p* Value)	DF	CFI	RMSEA	ΔChi-Square	Δdf	ΔCFI	ΔRMSEA	Decision
M1-configural	12.73	4	0.87	0.25	N/P	N/P	N/P	N/P	N/P
M2-metric	81.41	9	0.87	0.23	68.68	5	0	0.02	Accept (*p* = 0.47)
M3-scalar	166.34	17	0.73	0.24	84.93	8	0.01	0.01	Reject (*p* < 0.01)

## Data Availability

Data are available to all interested parties upon reasonable request.
